# Effects of a Follow-On Formula Containing Isomaltulose (Palatinose™) on Metabolic Response, Acceptance, Tolerance and Safety in Infants: A Randomized-Controlled Trial

**DOI:** 10.1371/journal.pone.0151614

**Published:** 2016-03-17

**Authors:** M. Fleddermann, A. Rauh-Pfeiffer, H. Demmelmair, L. Holdt, D. Teupser, B. Koletzko

**Affiliations:** 1 Dr. von Hauner Children`s Hospital, University of Munich Medical Centre, Munich, Germany; 2 Institute for Laboratory Medicine, University of Munich Medical Centre, Munich, Germany; Vanderbilt University, UNITED STATES

## Abstract

Effects of the dietary glycaemic load on postprandial blood glucose and insulin response might be of importance for fat deposition and risk of obesity. We aimed to investigate the metabolic effects, acceptance and tolerance of a follow-on formula containing the low glycaemic and low insulinaemic carbohydrate isomaltulose replacing high glycaemic maltodextrin. Healthy term infants aged 4 to 8 completed months (n = 50) were randomized to receive the intervention follow-on formula (IF, 2.1g isomaltulose (Palatinose™)/100mL) or an isocaloric conventional formula (CF) providing 2.1g maltodextrin/100mL for four weeks. Plasma insulinaemia 60min after start of feeding (primary outcome) was not statistically different, while glycaemia adjusted for age and time for drinking/volume of meal 60min after start of feeding was 122(105,140) mg/dL in IF (median, interquartile range) and 111(100,123) in CF (p = 0.01). Urinary c-peptide:creatinine ratio did not differ (IF:81.5(44.7, 96.0) vs. CF:56.8(37.5, 129),p = 0.43). Urinary c-peptide:creatinine ratio was correlated total intake of energy (R = 0.31,p = 0.045), protein (R = 0.42,p = 0.006) and fat (R = 0.40,p = 0.01) but not with carbohydrate intake (R = 0.22,p = 0.16). Both formulae were well accepted without differences in time of crying, flatulence, stool characteristics and the occurrence of adverse events. The expected lower postprandial plasma insulin and blood glucose level due to replacement of high glycaemic maltodextrin by low glycaemic isomaltulose were not observed in the single time-point blood analysis. In infants aged 4 to 8 completed months fed a liquid formula, peak blood glucose might be reached earlier than 60min after start of feeding. Non-invasive urinary c-peptide measurements may be a suitable marker of nutritional intake during the previous four days in infants.

***Trial registration*:** ClinicalTrials.gov NCT01627015

## Introduction

In infants significantly higher insulin levels were observed in formula-fed compared to breast-fed infants [1–4]. Insulin has been related to early weight gain [2], and higher insulin levels in formula-fed infants were found to be associated with higher risk of obesity and type 2 diabetes in later life compared to breast-fed infants [5,6]. Differences in long-term outcomes between breast- and formula-fed infants can be related to e.g. the different protein content [7] but also to the carbohydrate content of human milk and formula and its glycaemic and insulinaemic properties [8]. A study investigating the glycaemic index (GI) of various commercial infant and follow-on formulae with comparable protein composition in adults showed that the GI was higher in a formula containing high glycaemic maltodextrin in addition to lactose, if compared to a formula containing higher proportions of lactose [8]. The replacement of conventional higher glycaemic carbohydrates (glycaemic index >70) such as maltodextrins by isomaltulose (Palatinose™, glycaemic index = 32, [9]) can lead to a follow-on formula with a lower glycaemic and insulinaemic response.

The disaccharide isomaltulose contains glucose and fructose in a α-1,6 glycosidic linkage is completely digestible in the small intestine [9,10], but due to this glycosidic linkage it is only slowly hydrolyzed by small intestinal disaccharidases. In adults, the resulting postprandial glycaemia and insulinaemia following isomaltulose ingestion are lower as compared to usual disaccharides, while a higher blood glucose level is sustained for a longer period of time [9,11].

For these physiological reasons, isomaltulose may be of interest for the use in infant foods to reduce the glycaemic load and plasma insulin response while still providing the full amount of energy. This study aimed to investigate the effect of a follow-on formula with isomaltulose partly replacing conventional higher glycaemic carbohydrates on postprandial insulinaemia and glycaemia in infants at the age of 4 to 8 completed months. These possible effects might have a long-term preventive potential as diet induced enhancement of insulin secretion in infancy associated with increased early weight gain as well as higher long-term risk of obesity and associated disorders.

## Subjects and Methods

### Randomization and ethical consideration

Double-blinded randomized allocation of infants to the study formulae was stratified for gender, and a block size of four was applied. A random allocation sequence was computer generated by the study sponsor. The blinded allocation was maintained for participants, supporting staff and investigators until all laboratory and data analyses had been performed.

The study was conducted according to the guidelines laid down in the Declaration of Helsinki and all procedures involving human subjects were approved by the Ethical Committee of the Medical Faculty of Ludwig-Maximilians-University of Munich, Germany (reference number 114–12). Written informed consent was obtained from all parents prior to study start after the experimental protocol had been explained to them in detail. The study was registered at Clinical Trials.gov prior to study start on 21.06.2012 (NCT01627015, registration name: Safety, Acceptance and Metabolic Effects in Infants Receiving a Novel Low Glycaemic Index Follow-on Formula).

### Study design

The randomized, double-blind, controlled study with parallel design was performed with two formula groups (IF, intervention formula, CF, conventional formula). Infants of mothers who chose to formula -feed their infants for reasons not related to this study were recruited from age 4 to 8 completed months and were randomized double-blinded into one of the two formula groups. In European and German legislation and recommendations, the introduction of follow-on formula is recommended when the infant has received complementary feeding (solids). The introduction of complementary feeding is recommended between the beginning of the 5^th^ and 7^th^ month of life [12,13]. Infants fully replaced their usual formula meals by the study formula for 28 days. Dietary intake (4-day food protocol), sleep patterns and tolerance parameters were assessed by questionnaires for four days at study start (first four days when infants were fully fed with study formula) and again for four consecutive days at study end. Food protocols were reviewed by study personal and were discussed with families during the interview. On day 29, spontaneous urine was collected using urine bags for infants (B. Braun Melsungen AG, Germany). At the same day, after an at least 3-hour fast, the infants consumed 100 to 120 mL of their assigned formula. At 60 min after start of feeding, a single capillary blood sample was obtained by heel prick.

A blood sample was not taken at baseline to limit the burden for the participating healthy infants. Given that the amount and the time of the last meal did not differ between the groups, it appears likely that fasting blood values were comparable between both groups.

Anthropometric measurement was done by experienced study personal with detailed and intensive training in anthropometry and all other procedures both at study start and again on day 29. Weight was determined with a Seca 336 scale (Seca, Hamburg, Germany) equipped with a measuring rod (Seca 232) for measuring recumbent length. Head circumference was measured with a tape (Seca 212). Skinfolds were measured using a Holtain caliper (Holtain Ltd, Crymych, UK) at the left body axis at four sites (triceps, biceps, subscapular and mid-thigh). All measurements were performed in duplicates and documented with an accuracy of 10 g for weight and 0.1 cm for length and head circumference as well as 0.2 mm for skinfolds. The equipment was regularly checked and calibrated to ensure accuracy of measurements.

SD scores (z scores) were calculated for the anthropometric results relative to the growth standards of the WHO for breast-fed children [14]. Body fat percentage was calculated via predictive skinfold equations according to Slaughter et al. 1988 [15].

The glycaemic load was estimated by multiplying food specific glycaemic index [16] and the amount of carbohydrate, divided by 100. The calculated glycaemic index of the study formulae in consideration of specific carbohydrate composition was 48 for IF and 63 for CF [17]. The glycaemic index of complementary food was categorized into fruit, cereal, cereal plus fruit, potato/rice/noodle with vegetable and meat/fish, vegetable only, potato/rice/noodle with meat/fish, potato/rice/noodle with vegetable, potato/rice/noodle, yogurt, juice, salty snack and sweet snack. The glycemic load of complementary food was calculated by multiplying food category specific glycaemic index [16] or calculated glycaemic index according to Dodd et al. 2011 [17] and the amount of carbohydrate, divided by 100.

### Laboratory procedures

On study day 29, capillary blood samples were obtained by heel prick 60 min after start of feeding of 100 to 120 mL formula. Plasma aliquots for insulin measurement (collected with Potassium-EDTA Microvette^®^ CB 300, Sarstedt, Germany) and urine samples (Urine Monovette, Sarstedt, Germany) were stored at -80°C and transported on dry ice to the Institute for Laboratory Medicine of the University of Munich, Germany to analyze plasma insulin and urinary c-peptide, creatinine and urea respectively.

Quantitative insulin analysis was performed by commercially available Mercodia Ultrasensitive Insulin ELISA kit (Mercodia AB, Sweden), a solid phase two-sided enzyme immunoassay. The detection limit reported by the manufacturer is 0.07 mU/L [18]. The intra assay variation, analyzed in a pretest using 10 μl sample volume, was 3.1% (CV). Haemolysed plasma had no significant influence on insulin results.

Capillary postprandial blood glucose was measured using HemoCue^®^ Glucose 201 analyser (HemoCue AB, Angelholm, Sweden) and glycosylated hemoglobin (HbA1c) using Siemens/Bayer DCA 2000+ Analyzer (Siemens Healthcare Diagnostics Inc., USA). Urine was collected and analyzed for creatinine and urea using the Beckman Coulter^®^ AU 5800 analysis system (Beckman Coulter) according to the manufacturers´ instructions. Urinary c-peptide was determined using the cobas^®^ 8000 analyzer (e 602 module, Roche) according to the manufacturers´ instructions.

### Study population

From Dec 2012 to Mar 2014, 51 infants were enrolled via two recruitment strategies: 1.) Parents were asked for their general interest to participate in a clinical study with their infant at the maternity ward of the University of Munich, Germany. If interested, they were contacted again after 4 months, shortly before the study age of infants and study procedures were explained; 2.) Parents were informed about the study via letters providing study information and contact data of the principal investigator. If interested, they contacted the study center via phone or e-mail. Eligible infants (age: 4 to 8 completed months, actual weight-for-age: 5^th^ to 95^th^ percentile [19]) had to be born apparently healthy from singleton pregnancies at 37 to 42 weeks of gestation. Infants with acute or chronic disease or with mothers having gestational diabetes or a chronic disease influencing growth or metabolism were excluded from study participation. At baseline, energy and carbohydrate intake from complementary feeding did not exceed 25% of total intake, respectively, which was calculated for each infant individually.

### Study diets

Study formulae were provided free of charge to families in 450 g boxes labelled with random numbers. The products were packed in identically designed boxes labelled with the same product name. Both infant formulae were developed and produced on behalf of BENEO GmbH in accordance with relevant EU directives of 2006 [20]. The formulae were identical in respect to their composition including energy, protein, carbohydrate and fat content (S1 Table). The only difference concerns the carbohydrate composition of both formulae, in that maltodextrin (2.1 g/100mL) in the conventional formula was replaced by isomaltulose, while the further carbohydrates contained in the formulae (lactose and starch) were kept unchanged. Isomaltulose has been categorized as “generally recognized as safe” (GRAS) in the USA and has been approved as a food ingredient under the Novel Food regulation in the European Union [9]. The taste and appearance of isomaltulose are similar to sucrose and the sweetness is about half of that of sucrose [10]. Maltodextrin is a non-sweet palatable substance [21] and a high-glycaemic digestible carbohydrate obtained by the partial hydrolysis of starch. A free-of-choice provision of complementary diet (vegetable, fruit or potato) was allowed to be fed in addition to the study formulae up to a maximum of 25% of energy and carbohydrate intake.

### Power calculation, data management and statistical analysis

Power calculation was based on a study by Lucas et al. [22] with postprandial insulin values of 125 pmol/L in bottle-fed and 45 pmol/L in breast-fed infants and a standard deviation of 93.4 pmol/L. The study assumed a power of 85% and 5% risk of α-error to detect a difference as statistically significant required studying 22 infants per formula group. Assuming a total loss to follow-up rate of up to 15%, 25 infants were enrolled into each formula arm.

Data were entered into a database using ABBYY^®^ FlexiCapture 10 (Europe GmbH, Munich, Germany). Statistical analyses were performed with Stata^®^/MP 11.0 (StataCorp LP, College Station, TX, USA). Results are presented as mean ± SD or medians and interquartile ranges (IQR, 25th and 75th percentile). Pearson chi-square test was used for statistical comparison of categorical data. Student´s t-test was used for normally distributed continuous variables and Mann-Whitney test for non-normally distributed continuous variables. Group independent spearman rank correlations were used for correlations between c-peptide:creatinine ratio and nutritional intake to identify non-invasive biomarkers for nutrition. Linear regression model was applied to estimate the effect of formula type on the anthropometric outcome and biochemical outcome, including infants age (not z scores), volume and time of test meal (blood results only) and the respective baseline value (anthropometry only). Adjusted parameters are presented as coefficient and 95% confidence interval.

The primary (insulinaemia at 60 min after start of formula feeding) and secondary (glycaemia at 60 min after start of formula feeding, acceptance, tolerance, anthropometry parameters, urinary C-peptide, glycosylated haemoglobin) outcomes were analyzed for intention-to-treat and per-protocol populations. In the intention-to-treat analysis, all randomized subjects that received study formula were considered. In the per-protocol analysis, only data from subjects complying with the predefined conditions were included (formula intake covering more than 75% of energy and carbohydrate intake during whole study period, and who were fasted for more than 3 hours before the test meal).

## Results

**Fig 1:**
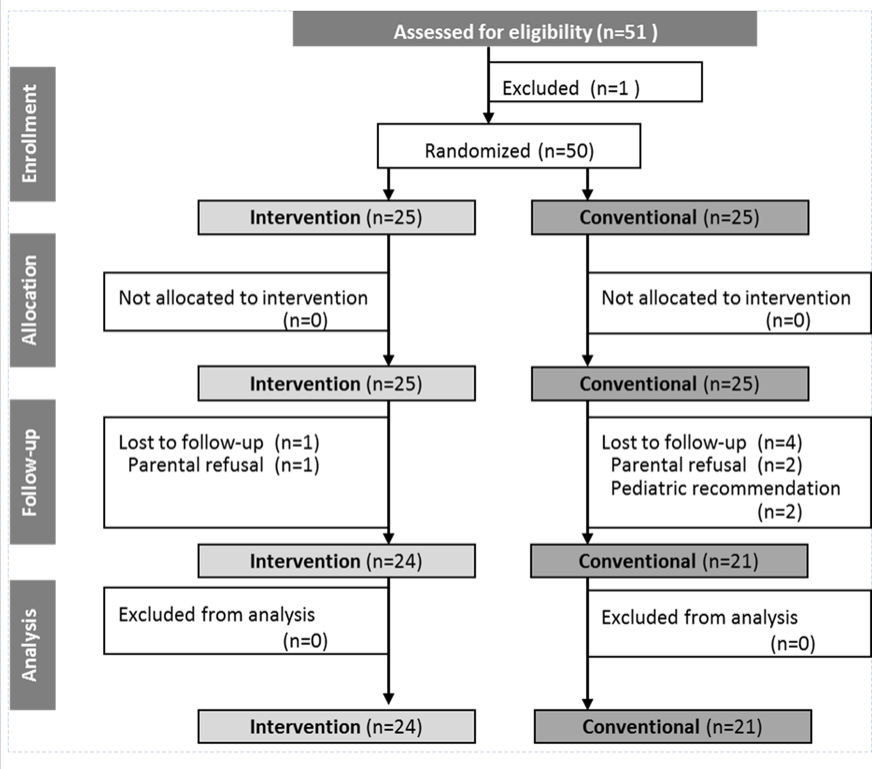
CONSORT chart. Numbers of participants for randomization, allocation, follow-up and analysis of low glycaemic intervention formula and conventional higher glycaemic formula group.

A total of 51 infants were recruited (Fig 1). Fifty infants were randomly allocated and received the study formulae from age 187 ± 40.0 days (IF) and 174 ± 30.2 (CF) days of life onwards (p = 0.17). During the intervention period five infants dropped out of the study (10%) because of parental refusal (n = 3: increased amount of flatulence n = 1, increased amount of flatulence plus firmer stool consistency n = 1, firmer stool consistency n = 1) or paediatric recommendation (n = 2: development of a bronchitis n = 1, firmer stool consistency n = 1), which were not statistically different between the groups. Gender ratio, birth order, socioeconomic data, nutritional intake and anthropometry at study entry (except subscapular skinfold thickness) were not significantly different between the randomized formula groups. At birth, small differences were observed for gestational age and anthropometry: IF infants were born at 40.1 weeks of gestation compared to 39.4 weeks for CF infants (p = 0.03). IF infants had mean birth weights and birth lengths of 3547 g and 52.6 cm, respectively, while CF infants had mean birth weights and birth lengths of 3329 g and 51.0 cm, respectively. Differences between the groups were statistically significant (p = 0.01) for birth length. However, at study entry, anthropometry parameters at the age of 4 to 8 completed months were not significantly different.

### Biochemical markers

All analyzed concentrations were within the normal ranges for healthy infants found in other studies (Table 1). Plasma insulin concentrations were not statistically different between the formula groups. Adjusted for age, time and volume of formula test meal, insulin concentration tended to be 2.60 μIU/mL higher in IF infants than CF infants (p = 0.10).

**Table 1:** Capillary postprandial plasma insulin, blood glucose and HbA1c and urinary c-peptide and urea concentrations at study end (intention-to-treat population).

	Intervention formula				Conventional formula				IF vs. CF^1^	Estimated difference at study end ^2^
	n	Median	IQR		n	Median	IQR		p value	
**Insulin** (μIU/mL)	24	8.66	(5.85;	11.9)	21	8.27	(5.72;	9.76)	0.39	-2.60 (-5.75, 0.55); p = 0.10
**Glucose** (mg/dL)	24	122	(105;	140)	21	111	(100;	123)	**0.048**	-15.8 (-26.8, -4.76); p = **0.006**
**HbA1c** (%)	24	4.90	(4.70;	5.15)	19	4.90	(4.80;	5.20)	0.96	0.06 (-0.16, 0.27); p = 0.60
**C-peptide** (ng/mL)	22	12.2	(6.70;	17.2)	21	13.0	(6.00;	19.3)	0.74	1.42 (-5.57, 8.41); p = 0.68
**C-peptide:creatinine** (ng/mg)	22	81.5	(44.7;	96.0)	21	56.8	(37.5;	129)	0.38	-14.1 (-49.6, 21.3); p = 0.43
**Urea** (g/L)	22	5.35	(4.50;	6.73)	21	6.27	(4.07;	7.94)	0.68	0.55 (-1.17, 2.28); p = 0.52
**Urea:creatinine**	22	36.2	(33.5;	42.4)	21	29.9	(27.5;	35.9)	**0.006**	-5.29 (-9.62, -0.95); p = **0.02**

Data presented as median (interquartile range). IF, intervention formula; CF, conventional formula; IQR, interquartile range.

^1^ Significant differences (Mann-Whitney test, P<0.05).

^²^ Derived from linear regression adjusted for age and time for drinking/ volume of meal (glucose and insulin only), 95% confidence interval in parentheses.

Capillary postprandial blood glucose levels were significantly higher in IF infants 122 (105,140) mg/dL (median (interquartile range)) compared to CF infants 111 (100,123) mg/dL (p = 0.048) in the intention-to-treat but not in the per-protocol population (123 (104,141) vs. 105 (100,123) mg/dL, p = 0.17). Adjusted for age, time and volume of formula test meal, postprandial blood glucose levels in IF infants were 15.8 mg/dL higher in IF than in CF infants (p = 0.006) in the intention-to-treat and tended to be higher in the per-protocol population (16.11 mg/dL higher in IF, p = 0.07). The percentage of glycosylated hemoglobin (HbA1c) did not differ between the formula groups with 4.90% in both groups (p = 0.96).

With respect to urine analysis, neither urinary c-peptide levels nor urinary c-peptide:creatinine ratio differed significantly between formula groups. The urinary c-peptide levels were directly related to weight gain (R = 0.51, p = 0.0004) and total energy, protein and fat intake of the previous four days (energy intake: R = 0.31, p = 0.045; protein intake: R = 0.42, p = 0.006; fat intake R = 0.40, p = 0.01; but not of carbohydrate intake: R = 0.22, p = 0.16). The urinary urea concentration did not differ significantly between the formula groups, while the urea:creatinine ratio was significantly higher in IF infants 36.2 (33.5, 42.4) than in CF 29.9 (27.5,35.9) infants (p = 0.006).

### Acceptance, tolerance and adverse events

Both formulae were well-accepted. No significant differences were observed for acceptance as well as tolerance parameters, such as regurgitation, vomiting, duration of crying and sleeping duration (data not shown). The amount of flatulence was generally low and was not significantly different between groups at study entry (first four days fully fed by study formula). During the second half of the study the average amount of flatulence tended to be higher in CF infants than in IF infants (S2 Table). Stool frequency and stool consistency did not differ among the two formula groups.

Adverse events were observed in 19 IF and 18 CF infants (p = 0.75). The types of adverse events were similarly distributed between formula groups. Gastrointestinal events were partly associated with other illnesses and did not differ between the groups. The coherence with formula and intensity of adverse events did not differ (Table 2). Only one serious adverse event was recorded during the study in the CF group, which was not formula-related.

**Table 2:** Summary of Adverse events.

	Intervention formula	Conventional formula	IF vs. CF^1^
	n	n	p value
**No of infants**	25	25	
**All SAE**	0	1*	0.31
**No of infants with minimum 1 NSAE**	19	18	0.75
**All NSAE**	26	24	
**NSAE-gastrointestinal**	14	12	0.79
**NSAE-skin**	2	1	0.55
**NSAE-infection**	4	4	1.00
**NSAE-respiratory**	6	7	0.75
**NSAE-coherence with formula** (no/weak/likely/possible)	13/8/1/4	12/7/2/3	0.63
**NSAE-intensity** (mild/moderate/severe)	13/11/2	14/7/3	0.37
**Dropout rate**	1	4	0.157

IF, intervention formula; CF, conventional formula; NSAE, non-serious adverse events; SAE, serious adverse events.

^1^ Significant differences (Chi-square test, P<0.05).

* Non formula related event

### Growth

Anthropometric data including skinfolds and z scores determined at baseline and study end are summarized in Table 3. No statistical differences were observed at baseline except for subscapular skinfold thickness, that was 7.63 ± 1.38 mm (IF) and 6.86 ± 1.30 mm (CF, p = 0.049), and respective calculated body fat content [15] of 19.0 ± 2.77% and 17.3 ± 2.78% (p = 0.04). Non-adjusted weight gain was not statistically different between formula groups (IF: 14.2 ± 5.74 vs. CF: 11.0 ± 5.98 g/d, p = 0.08). Length gain expressed as mm per day was not different between the groups (IF: 0.55 ± 0.27 mm/d and CF: 0.55 ± 0.22 mm/d, p = 0.95). Adjusted for the respective anthropometric baseline value and age, weight gain was 3.19 g/d non-significantly higher in IF than in CF infants (p = 0.06). Adjusted length gain was not different (0.05 mm/d higher in IF infants than in CF infants, p = 0.49). No differences were observed for head circumference gain non-adjusted and adjusted. Analyzing per-protocol subjects, weight gain was not statistically different between the groups (IF: 14.2 ± 5.38 and CF: 12.1 ± 5.41 g/d, p = 0.31). Length gain and head circumference gain did not differ, as well.

**Table 3:** Number of children, age at measurement and anthropometric measures with z scores at baseline and study end.

	Intervention formula		Conventional formula		P value^1^	Estimated difference at study end ^²^
	Baseline	Study end	Baseline	Study end	IF *vs*. CF	
**No. of children**	25	24	25	21		
**Age** (d)	187 ± 40.0	223 ± 39.5	174 ± 30.2	207 ± 29.5	0.14	-0.18 (-1.55, 1.18); p = 0.79
**Sex** (male/female)	13/12	12/12	14/11	11/10		
**Weight**						
(g)	7937 ± 868	8434 ± 964	7560 ± 791	7825 ± 794	**0.03**	-101 (-222, 19.5); p = 0.10
(z score)	0.24 ± 0.82	0.32 ± 0.95	-0.01 ± 0.79	0.15 ± 0.84	0.08	-0.08 (-0.20, 0.03); p = 0.16
**Length**						
(cm)	67.6 ± 2.63	69.5 ± 2.49	66.7 ± 2.46	68.2 ± 2.23	0.09	-0.20 (-0.70, 0.30); p = 0.43
(z score)	0.28 ± 0.86	0.29 ± 0.93	0.19 ± 0.89	0.07 ± 0.79	0.41	-0.06 (-0.28, 0.16); p = 0.60
**Head**						
(cm)	43.1 ± 1.46	43.9 ± 1.54	43.0 ± 1.42	43.9 ± 1.08	0.86	0.30 (-0.09, 0.68); p = 0.13
**BMI**						
(z score)	0.12 ± 0.76	0.21 ± 0.94	-0.16 ± 1.08	-0.27 ± 1.24	0.14	-0.09 (-0.34, 0.15), p = 0.45
**Weight-for-length**						
(z score)	0.20 ± 0.76	0.30 ± 0.93	-0.09 ± 1.10	-0.19 ± 1.22	0.13	-0.10 (-0.33, 0.14); p = 0.42
**Circumferences**						
Upperarm (cm)	15.0 ± 1.02	15.5 ± 1.33	14.7 ± 1.18	14.8 ± 1.16	0.08	-0.16 (-0.49, 0.16); p = 0.32
Chest (cm)	44.3 ± 2.15	45.2 ± 2.47	43.2 ± 1.83	42.5 ± 4.97	**0.02**	-1.04 (-2.99, 0.90); p = 0.28
Waist (cm)	42.5 ± 2.68	43.5 ± 2.87	41.6 ± 2.32	41.8 ± 2.67	**0.048**	-0.35 (-1.19, 0.49); p = 0.41
Thigh (cm)	24.2 ± 1.91	24.9 ± 2.42	23.7 ± 1.91	23.9 ± 2.13	0.14	-0.10 (-0.77, 0.56); p = 0.76
**Skinfolds**						
Triceps (mm)	12.4 ± 2.68	11.8 ± 2.18	11.2 ± 2.17	11.2 ± 2.62	0.35	0.21 (-0.85, 1.27); p = 0.69
Biceps (mm)	7.04 ± 1.66	6.65 ± 1.66	6.95 ± 1.32	6.78 ± 1.63	0.78	0.31 (-0.50, 1.12); p = 0.45
Subscapular (mm)	7.63 ± 1.38	7.67 ± 2.12	6.86 ± 1.30	6.63 ± 1.65	0.08	-0.42 (-1.56, 0.73); p = 0.73
Midthigh (mm)	20.7 ± 4.53	19.6 ± 4.10	20.0 ± 3.15	20.0 ± 4.31	0.73	0.71 (-0.78, 2.20); p = 0.34
**Body fat (%)** ^3^	19.0 ± 2.77	18.5 ± 2.77	17.3 ± 2.78	17.0 ± 3.40	0.09	-0.22 (-1.74, 1.30); p = 0.77

IF, intervention formula; CF, conventional formula. Data presented as mean ± standard deviation.

^1^ Significant differences (Student´s t-test, P<0.05) at study end.

^2^ Derived from linear regression adjusted for the respective anthropometric baseline value and age (not z score), 95% confidence interval in parentheses.

^3^ Calculated by Slaughter et al. 1988 [15].

### Nutritional intake

Energy intake from formulae determined from the 4-day protocols (S3 Table) was identical in IF and CF infants at study start and end (study start: 578 ± 123 vs. 549 ± 104 kcal/d, p = 0.39 and study end: 562 ± 154 and 541 ± 96.4 kcal/d, p = 0.58). Adjusted for age, energy intake did not differ between the groups, as well. A total of 43 infant consumed complementary food during the study. The mean energy intake from complementary feeding was 108 ± 69.5 kcal/d in IF infants and 104 ± 84.3 kcal/d in CF infants (p = 0.86) at study start and 124 ± 87.5 kcal/d and 112 ± 96.0 kcal/d (p = 0.67) for the IF and CF infants at study end, respectively. Carbohydrate intake was 15.0 ± 10.3 g/d (IF) and 13.8 ± 12.2 g/d (CF) at study start (p = 0.75) and 17.7 ± 13.2 g/d and 16.0 ± 16.7 g/d (p = 0.70) at study end, respectively. Protein intake and fat intake from complementary feeding did not differ. The glycaemic load via complementary feeding did not differ between the groups and ranged between 8.71 ± 7.88 and 12.8 ± 10.9 per day in infants aged 4 to 8 completed months. There were no significant differences between the intake of water, tea or other formula than study formula, between the formula groups during the whole study period. The frequency of formula meals, complementary feeding and total meals was not different between both groups. The total number of meals per day was nearly constant during the study period (6.20 to 6.39 meals/d), number of formulae meals slightly decreased (from 5.07 to 4.75 meals/d) while complementary feeding meals increased (1.41 to 1.69 meals/d). In the per-protocol population, no differences in energy intake from formulae, energy and macronutrient intake from complementary feeding were found similar like in the intention-to-treat population. Neither intake of liquid nor meals per day were different between IF and CF infants (data not shown).

## Discussion

### Determination of postprandial insulin and glucose in infants

In our randomized, controlled, double-blind intervention study, postprandial plasma insulin levels assessed 60 min after start of feeding did not differ between the formulae groups. In contrast, the measurement of postprandial glycaemia at a single time point 60 min after the start of feeding resulted in significantly higher levels in IF than in CF infants. The insulin response is directly related to glucose response [9,23]. Other studies [9,24] reported reduced postprandial glucose and insulin levels after isomaltulose compared to sucrose administration in human adults and in animals [9,11,24,25]. None of these studies was performed in infants or used maltodextrin as control. Our blood glucose and plasma insulin levels were assessed at a single time point only. In published literature, isomaltulose as low glycaemic carbohydrate resulted in a lower blood glucose response, when determined as area under the curve [9]. Isomaltulose has a lower glycaemic index than maltodextrin. This is on the one hand due to the slower digestion of isomaltulose and on the other hand, due to the monosaccharide composition: isomaltulose (glucose and fructose) *vs*. maltodextrin (glucose only), whereas fructose has a lower glycaemic index than glucose [16]. The slower release and absorption of the derived monosaccharides lead to lower postprandial glycaemia and insulinaemia than after oral sucrose administration, and the blood glucose level is sustained for a longer period of time [9,11]. Thus from about 90 min onwards, slightly enhanced glucose levels were observed in isomaltulose groups [9,23,25,26]. Most of these studies were performed in adults or animals and sucrose was the control carbohydrate.

Data on the postprandial blood glucose and insulin responses to formula feeding in infants are limited. Lucas et al. [1] report postprandial insulin values in breast-fed and formula-fed infants peaking at 55 and 90 min, respectively. Similarly, Salmenpära et al. [3] reported in infants aged 9 months glucose and insulin levels that peaked between 55 and 90 min after the beginning of an ad libitum feeding. Practical and ethical issues exist performing feeding tests in infants and collecting frequent capillary blood samples as recently also acknowledged by Wright et al. 2015 [8]. Due to such practical and ethical issues and to minimize the burden and inconvenience to the infants, postprandial blood sampling in our infant trial was limited to a single time point only. Based on the above cited published literature we decided to collect capillary blood samples at 60 min after start of feeding [22]. Thus there is the possibility that the chosen time point for postprandial blood glucose measurement at 60 min after start of feeding in our study was in a cross-over period when blood glucose with the IF formula containing low glycaemic isomaltulose was still increasing and levels with the CF formula containing high glycaemic maltodextrin were already decreasing. Early peaks of glycaemia may not have been detected if blood is taken 55 or 60 min after beginning of feeding [27] and thus higher glucose levels resulting from delayed digestion of isomaltulose may have been detected. The availability of more data about postprandial glucose response in infants over a longer time is warranted in order to benefit the general understanding of metabolic processes in this age group.

The glucose and insulin response depend on the composition of the formula, but also on the absolute amount of carbohydrates consumed. Moreover, also the time needed to consume the respective formula volume could influence the postprandial response. The average amount of formula consumed at the study day 60 min before blood sampling was similar with 114±7.61 mL in the IF group and 114±6.15 mL in the CF group (p = 0.78). The average time needed to consume the respective formula portion was 10.1±7.57 min in the IF group and 7.29±4.17 min in the CF group (p = 0.13). To account for different drink volumes and thus carbohydrate amounts consumed as well as for the time infants needed to consume the formula, we adjusted the postprandial blood glucose and insulin levels for volume of formula test meal as well as time needed.

### HbA1c, c-peptide and nutritional intake

HbA1c is used for glycaemic control from childhood onwards and reflects mean plasma glucose during the past 1 to 2 months [28]. In young infants, HbA1c is critically discussed, due to the influence of fetal hemoglobin that falsely increases HbA1c values [28]. The content of fetal hemoglobin decreases during the first year of life: at birth 80–90% accounts for fetal hemoglobin, at the age of 6 months largest proportion of hemoglobin is hemoglobin A (adult hemoglobin) and at the age of 1 year fetal hemoglobin decreases to less than 1% [28]. At our study age of about 6 months, HbA1c values did not differ between the formulae groups (4.90% in both groups) after an intervention period of 1 month. These values were within the reference ranges of 6 months and 8 to 12 months old infants [28,29] of 4.80 to 6.00%.

Urinary c-peptide as marker of endogenous insulin secretion in adults [27,30,31] was associated with infant weight gain in our study, which was comparable to the findings of another trial in infants aged 5 to 9 months [2]. Good correlations were found for urinary c-peptide levels and nutritional intake of the previous four days. The urinary c-peptide measurements might be a suitable and non-invasive marker of insulin secretion [32] and of the nutritional intake that can be used to evaluate a metabolic response by especially younger study participants that is further well tolerated. Total energy intake, as well as fat and protein intake were related with urinary c-peptide:creatinine ratio. The carbohydrate intake was not related to c-peptide:creatinine ratio. While higher carbohydrate intake has increased insulin secretion, the lower glycaemic carbohydrate isomaltulose could have lowered the insulin secretion [9] and thus both factors might have acted contrary. Strongest correlation was found for protein intake and urinary c-peptide in our study. Similar results were found in a multicenter study in which 577 infants received either a high or a lower protein formula, and serum c-peptide levels were found positively related with protein intake [33]. C-peptide as metabolic part of the insulin-IGF-axis has a role for early programming. High insulin-IGF blood concentrations in early infancy, as a result of infant formula composition e.g. higher protein content [33], increase BMI by 0.51 (0.13,0.90; p = 0.009) at school age and increase the risk for obesity [34].

In infants aged 4 to 8 completed months, the total energy intake was 634 to 676 kcal per day and complementary feeding covered about 15.6 to 18.2% of total energy intake, which are typical of infants in this age group in Germany [35]. No compensatory increase of glycaemic load via complementary feeding was observed in IF group, which ranged between 8.71 and 12.8 and did not differ significantly between the groups. The total numbers of meals per day was about 6.3, with about 4.9 formula meals per day. Although isomaltulose markedly regulate satiety-regulating peptides, when administered alone [11], the whole macronutrient composition of a meal might be more important in regulation of satiety response [11].

### Acceptance and tolerance

This study demonstrated that the acceptance and tolerance of a follow-on formula with isomaltulose was similar to that of conventional follow-on formulae. All infants accepted the isomaltulose formula well. No negative effects were found for the number of adverse events, the amount of flatulence, stool consistency and stool frequency.

Infants aged 4 to 8 completed months are in a sensitive phase of growth and development. The gastrointestinal burden is high due to the ongoing development of the gut being exposed to a variety of different foods for the first time [36]. Isomaltulose was a well-digestible carbohydrate also for this age group without negatively influencing normal laxation. From literature it is known, that disaccharidase activities in infants aged about 180 days (11.2 units- micromoles of substrate hydrolyzed per minute at 37°C per gram of protein) is similar to that in older children (12.7 units) [37].

### Strengths and limitations

The strength of the study was, that stool parameters, flatulence, regurgitation and vomiting, were assessed high frequently (four days in the beginning and four days at the end of the study) which was useful for evaluation of acceptance and tolerance in infants. Further, except for the carbohydrate source that was partly replaced by isomaltulose, all other ingredients and contents remained identical in both formulae. However, we cannot exclude, that the modified carbohydrate composition might have an influence on metabolic processes as fat oxidation, which might influence fat mass deposition [38–40].

A limitation of this study in infants was the blood sampling at a single time point only. Number of blood samplings for each individual was limited to a minimum for ethical reasons. Such ethical and practical constraints of feeding test foods to infants and collecting frequent capillary blood samples have also recently been acknowledged by Wright et al. 2015 [8]. Defining the exact time point for capillary withdrawal at 60 min after start of feeding was based on literature data [22], and might limit the conclusions that can be drawn from this study. Less data were available about postprandial glucose and insulin curves in infants. Only a small amount of markers to evaluate metabolic processes in infants are available. The general markers, require special conditions as a fasting state, are less applicable in this age group. Thus e.g. HOMA index (Homeostasis Model Assessment) to evaluate insulin sensitivity could not be used or connections e.g. between blood insulin and urinary c-peptide were not found in our study. The development of new age-appropriate investigation procedures and the application of new markers to gain further knowledge about infant metabolism and possible later outcomes have to be examined.

In conclusion, metabolic differences such as the expected reduced postprandial blood glucose and plasma insulin response were not observed. This may be due to the limitations of the trial conditions in this age group (in particular blood collection at a single time point only), rather than the absence of difference in view of the established differences in insulin and glucose response between maltodextrin and isomaltulose. Non-invasive urinary c-peptide measurements might be a suitable marker of nutritional intake during the previous four days in infants aged 4 to 8 completed months. Both follow-on formulae were tolerated well in infants aged 4 to 8 completed months. Follow-on formulae containing isomaltulose was well accepted.

## Supporting Information

S1 Table Nutritional composition of study follow-on formulae. (DOCX)

S2 Table Number of infants with respective stool consistency, stool frequency (number of stools per day) and amount of flatulence (hours per day) during a four day period at study start and end. (DOCX)

S3 Table Daily nutritional intake of infants aged 4 to 8 completed months (intention-to-treat population). (DOCX)

S1 Text Study protocol. (PDF)

S2 Text Amendments to the study protocol. (PDF)

S3 Text CONSORT checklist. (DOC)

## Abbreviations

IF: intervention formula
CF: conventional formula
