# Supplementary material for: Effects of a Follow-On Formula Containing Isomaltulose (Palatinose™) on Metabolic Response, Acceptance, Tolerance and Safety in Infants: A Randomized-Controlled Trial
Source: PLoS One. 2016 Mar 17;11(3):e0151614. doi: 10.1371/journal.pone.0151614 (PMC4795687; doi:10.1371/journal.pone.0151614)
Supplement: S1 Table — (DOCX) [file pone.0151614.s001.docx]

S1 Table Nutritional composition of study follow-on formulae.

|  | **Intervention formula** | | | |  | **Conventional formula** | | | |  | |
| --- | --- | --- | --- | --- | --- | --- | --- | --- | --- | --- | --- |
|  |  | g/ 100mL |  | |  |  | g/ 100mL |  | |  | |
| **Energy** (kcal) |  | 70 |  |  |  |  | 70 |  |  |  |  |
| **Protein**  Whey protein:casein |  | 1.9  42:58 |  |  |  |  | 1.9  42:58 |  |  | |  |
| **Fat** |  | 3.3 |  |  |  |  | 3.3 |  |  | |  |
| **Carbohydrates** |  | 8.3 |  |  |  |  | 8.3 |  |  | |  |
| Lactose |  | 5.3 |  |  |  |  | 5.3 |  |  | |  |
| Maltodextrin |  | - |  |  |  |  | 2.1 |  |  | |  |
| Starch |  | 0.9 |  |  |  |  | 0.9 |  |  | |  |
| Isomaltulose |  | 2.1 |  |  |  |  | - |  |  | |  |

For the calculation of glycaemic indices of study formulae: glycaemic index of 46 for lactose, 90 for starch and maltodextrin and 32 for isomaltulose was used.
