# Supplementary material for: Effects of a Follow-On Formula Containing Isomaltulose (Palatinose™) on Metabolic Response, Acceptance, Tolerance and Safety in Infants: A Randomized-Controlled Trial
Source: PLoS One. 2016 Mar 17;11(3):e0151614. doi: 10.1371/journal.pone.0151614 (PMC4795687; doi:10.1371/journal.pone.0151614)
Supplement: S2 Table — (DOCX) [file pone.0151614.s002.docx]

S2 Table Number of infants with respective stool consistency, stool frequency (number of stools per day) and amount of flatulence (hours per day) during a four day period at study start and end.

|  |  | **Intervention formula** | |  | **Conventional formula** | |  | **p value** (IF *vs.* CF) ^1^ | |
| --- | --- | --- | --- | --- | --- | --- | --- | --- | --- |
|  |  | Study day  1-4 | Study day  25-28 |  | Study day  1-4 | Study day  25-28 |  | Study day  1-4 | Study day  25-28 |
| **Stool consistency** | Watery | 0 | 0 |  | 0 | 0 |  | 0.31 | 0.09 |
|  | Thin | 4 | 1 |  | 6 | 3 |  |  |  |
|  | Pasty | 55 | 48 |  | 50 | 52 |  |  |  |
|  | Viscous | 20 | 34 |  | 18 | 15 |  |  |  |
|  | Firm | 12 | 9 |  | 9 | 4 |  |  |  |
|  |  |  |  |  |  |  |  |  |  |
| **Stool frequency (n per day)** | 0 | 3 | 3 |  | 2 | 1 |  | 0.87 | 0.91 |
|  | 1-2 | 69 | 63 |  | 66 | 58 |  |  |  |
|  | 3 | 18 | 23 |  | 18 | 18 |  |  |  |
|  | 4-5 | 6 | 6 |  | 5 | 6 |  |  |  |
|  | >5 | 0 | 0 |  | 0 | 0 |  |  |  |
|  |  |  |  |  |  |  |  |  |  |
| **Flatulences (hour per day)** | 0 | 72 | 81 |  | 56 | 50 |  | 0.90 | **0.04** |
|  | <1 | 21 | 15 |  | 29 | 33 |  |  |  |
|  | 1-2 | 2 | 0 |  | 5 | 1 |  |  |  |
|  | 3-4 | 0 | 0 |  | 0 | 0 |  |  |  |
|  | >4 | 0 | 0 |  | 0 | 0 |  |  |  |

Number of infants record the respective stool amount, consistency or amount of flatulence during the four day period at study start (study day 1-4) or end (study day 25-28). IF, intervention formula; CF, conventional formula. ^1^ Significant differences (Mann-Whitney test, P<0.05).
