# Supplementary material for: Effects of a Follow-On Formula Containing Isomaltulose (Palatinose™) on Metabolic Response, Acceptance, Tolerance and Safety in Infants: A Randomized-Controlled Trial
Source: PLoS One. 2016 Mar 17;11(3):e0151614. doi: 10.1371/journal.pone.0151614 (PMC4795687; doi:10.1371/journal.pone.0151614)
Supplement: S3 Table — (DOCX) [file pone.0151614.s003.docx]

S3 Table Daily nutritional intake of infants aged 4 to 8 completed months (intention-to-treat population).

|  | Study start | | | | | | | |  | Study end | | | | | | | |
| --- | --- | --- | --- | --- | --- | --- | --- | --- | --- | --- | --- | --- | --- | --- | --- | --- | --- |
|  | **Intervention formula** | | |  | **Conventional formula** | | | **IF** *vs.* **CF** |  | **Intervention formula** | | |  | **Conventional formula** | | | **IF** *vs.* **CF** |
|  | n | Mean | SD |  | n | Mean | SD | p value |  | n | Mean | SD |  | n | Mean | SD | p value |
|  |  |  |  |  |  |  |  |  |  |  |  |  |  |  |  |  |  |
| **Age of infant (d)** | 24 | 194 | 39.2 |  | 23 | 178 | 28.2 | 0.12 |  | 24 | 220 | 39.5 |  | 21 | 204 | 29.4 | 0.14 |
| **No. of meals per day (n)** | 24 | 6.2 | 1.16 |  | 23 | 6.29 | 1.47 | 0.8 |  | 24 | 6.36 | 1.24 |  | 21 | 6.2 | 1.58 | 0.7 |
| **Total energy intake (kcal)** | 24 | 663 | 115 |  | 23 | 634 | 109 | 0.38 |  | 24 | 676 | 158 |  | 21 | 652 | 98.3 | 0.56 |
| **Total glycaemic load** | 24 | 39.8 | 8.22 |  | 23 | 46.9 | 8.23 | 0.005 |  | 24 | 42.9 | 11.6 |  | 21 | 48.0 | 8.54 | 0.11 |
| **No. formula meals per day (n)** | 24 | 5.07 | 1.20 |  | 23 | 4.90 | 1.31 | 0.64 |  | 24 | 4.89 | 1.41 |  | 21 | 4.75 | 1.41 | 0.75 |
| **Volume of formula (mL)** | 24 | 808 | 186 |  | 23 | 776 | 155 | 0.53 |  | 24 | 787 | 231 |  | 21 | 759 | 141 | 0.63 |
| Energy (kcal) | 24 | 578 | 123 |  | 23 | 549 | 104 | 0.39 |  | 24 | 562 | 154 |  | 21 | 541 | 96.4 | 0.58 |
| Protein (g) | 24 | 14.0 | 2.99 |  | 23 | 13.3 | 2.52 |  |  | 24 | 13.6 | 3.72 |  | 21 | 13.1 | 2.33 |  |
| Carbohydrates (g) | 24 | 66.4 | 14.2 |  | 23 | 63.1 | 12.0 |  |  | 24 | 64.6 | 17.7 |  | 21 | 62.1 | 11.1 |  |
| Fat (g) | 24 | 28.0 | 5.97 |  | 23 | 26.5 | 5.03 |  |  | 24 | 27.2 | 7.43 |  | 21 | 26.2 | 4.66 |  |
| Glyaemic load via study formulae | 24 | 31.9 | 6.81 |  | 23 | 39.7 | 7.53 | 0.0005 |  | 24 | 31.0 | 8.47 |  | 21 | 39.1 | 6.98 | 0.001 |
| **No. complementary meals per day (n)^1^** | 15 | 1.53 | 0.52 |  | 16 | 1.41 | 0.54 | 0.51 |  | 20 | 1.69 | 0.57 |  | 14 | 1.57 | 0.82 | 0.63 |
| **Amount complementary food (g)** | 19 | 170 | 121 |  | 19 | 148 | 105 | 0.56 |  | 22 | 195 | 133 |  | 21 | 163 | 136 | 0.44 |
| Energy (kcal) | 19 | 108 | 69.5 |  | 19 | 104 | 84.3 | 0.86 |  | 22 | 124 | 87.5 |  | 21 | 112 | 0.67 | 0.67 |
| Protein (g) | 19 | 3.02 | 2.30 |  | 19 | 3.11 | 2.94 | 0.91 |  | 22 | 3.87 | 2.92 |  | 21 | 2.92 | 2.61 | 0.27 |
| Carbohydrates (g) | 19 | 15.0 | 10.3 |  | 19 | 13.8 | 12.2 | 0.75 |  | 22 | 17.7 | 13.2 |  | 21 | 16.0 | 16.7 | 0.7 |
| Fat (g) | 19 | 3.77 | 2.92 |  | 19 | 3.73 | 3.38 | 0.96 |  | 22 | 3.99 | 3.29 |  | 21 | 3.64 | 2.97 | 0.71 |
| Glyaemic load via complementary food | 19 | 10.0 | 7.73 |  | 19 | 8.71 | 7.88 | 0.61 |  | 22 | 12.8 | 10.9 |  | 21 | 8.82 | 8.88 | 0.18 |
| Percent of energy intake (%) | 19 | 16.3 | 10.0 |  | 19 | 15.6 | 11.8 | 0.85 |  | 22 | 18.2 | 10.8 |  | 21 | 16.4 | 12.3 | 0.62 |
| Percent of carbohydrate intake (%) | 19 | 18.6 | 11.9 |  | 19 | 17.3 | 13.2 | 0.75 |  | 22 | 21.3 | 13.3 |  | 21 | 18.5 | 14.8 | 0.52 |
| **Amount liquid (mL)** | 5 | 34.0 | 23.0 |  | 10 | 44.0 | 20.7 | 0.41 |  | 7 | 25.2 | 12.7 |  | 11 | 50.2 | 68.8 | 0.36 |
|  |  |  |  |  |  |  |  |  |  |  |  |  |  |  |  |  |  |

Data presented as mean and standard deviation. IF, intervention formula; CF, conventional formula; SD, standard deviation; ITT, intention-to-treat. Significant differences (Student´s t-test, P<0.05).

^1^ Kind of meal was sorted according to weight proportion, e.g. 30 g of complementary food plus 200 mL milk was sorted as milk meal.

^2^ Glycemic load was calculated by multiplying food category specific glycaemic index ([16](#_ENREF_16)) or calculated glycaemic index according to Dodd et al. 2011 ([17](#_ENREF_17)) and the amount of carbohydrate, divided by 100.
