# Supplementary material for: Effects of a Follow-On Formula Containing Isomaltulose (Palatinose™) on Metabolic Response, Acceptance, Tolerance and Safety in Infants: A Randomized-Controlled Trial
Source: PLoS One. 2016 Mar 17;11(3):e0151614. doi: 10.1371/journal.pone.0151614 (PMC4795687; doi:10.1371/journal.pone.0151614)
Supplement: S1 Text — (PDF) [file pone.0151614.s004.pdf]

Safety, acceptance and metabolic effects in infants receiving a novel low glycaemic index follow-on formula

Short title: **AMELIE**, **A**cceptance and **Me**tabo**l**ism of **I**somaltulos**e**

Div. Metabolic Diseases and Nutritional Medicine (Head. Prof. Berthold Koletzko), Dr. von Hauner Children's Hospital, Ludwig-Maximilians-University Munich, Germany

and BENEO GmbH, Wormser Str. 11, 67283 Obrigheim/Pfalz

1. Summary 2
2. Investigators and financial support 2
3. Introduction 3
4. Objectives 4
5. Participating subjects 5
6. Study Procedure 6
7. Study supplement 10
8. Laboratory analyses 11
9. Data management and biometrical evaluation 11
10. Ethical consideration, safety and insurance 12
11. References 13

1. Summary

Significant differences of insulinemia between breast fed and formula fed infants have been observed. Insulin has been related to early growth and breast-feeding was found to be associated with lower risk of obesity and Type 2 diabetes in later life compared with formula feeding. Thus, effects of the glycaemic index of the meal on plasma glucose and insulin concentrations during the postprandial period might be of importance. The disaccharide isomaltulose contains glucose and fructose in α-1,6 glycosidic linkage and is due to this bond only slowly, but completely hydrolyzed by small intestinal disaccharidases resulting in less postprandial glucose and insulin increase and glucose supply is sustained for a longer period of time. This study aims to investigate the acceptance, tolerance, and the effect on postprandial glycaemia and insulinemia of a follow-on formula with isomaltulose (Palatinose™) replacing conventional higher glycaemic carbohydrates in comparison to a standing formula with otherwise identical composition. In total 50 infants at the age of 5 - 7th month of life shall be enrolled and consume one of the both study formulae during a 4 weeks intervention period with detailed recording of infantile diet, health, and behaviour. Postprandial glucose and insulin measurement will be conducted at the end of the intervention period. Study results will enable to discuss the potential of isomaltulose as a low glycaemic component in infant formula.

2. Investigators and financial support

Principal investigator:

Professor Berthold Koletzko
Div. Metabolic Diseases and Nutritional Medicine
Dr. von Hauner Children's Hospital, Ludwig-Maximilians-University of Munich
Lindwurmstr. 4, D-80337 Muenchen
Tel: +49 (0)89 5160 2826, Fax: +49 (0)89 5160 7742
E-mail: office.koletzko@med.uni-muenchen.de

Co-investigators:

Prof. Vogeser

TBD

Sponsor:

The study is part of the EU-project "Early Nutrition". This part of the project is sponsored by:

BENEO GmbH, Wormser Str. 11, 67283 Obrigheim/Pfalz

Contact person on behalf of the sponsor will be

Ines Holub telephone: 06359 / 803-528
fax: 06359 / 803-153
e-mail: ines.holub@beneo.com

3. Introduction

The glycaemic index of a carbohydrate rich meal is defined by the postprandial increase of blood glucose concentrations compared to a standard food. Glycaemic index and amount of carbohydrate are the major determinants of the glycaemic load associated with a meal or a habitual diet, respectively. Observational studies in adults suggest that diets with a high glycaemic load are associated with a higher risk of type 2 diabetes (Zhang, Liu et al. 2006) and cardiovascular diseases (Oh, Hu et al. 2005). The high glycaemic response after consumption of a high glycaemic meal is followed by an immediate high insulinaemic response. In contrast, a lower glycaemic response after consumption of a low glycaemic meal leads to a low insulinaemic response.

In infants significant differences of the insulinemia between breast fed and formula fed infants have been observed. Very young infants aged only about one week showed lower postprandial increases in insulin following breast feeding (Lucas, Boyes et al. 1981) and even at the age of nine months, when complementary feeding was already an important component of the diet breast fed infants had significantly lower insulin concentrations 150 min after their feeding than infants not breast fed (Madsen, Schack-Nielsen et al. 2010). While no marked differences in postprandial glucose between formula and breast milk were observed in two studies, postprandial insulin was lower in 9 months old breast fed infants compared to formula fed infants (Salmenpera, Perheentupa et al. 1988) and 3-6 month old breast fed infants had significantly lower postprandial serum c-peptide than the formula fed infants (Wallensteen, Lindblad et al. 1991). This different metabolic reaction has received considerable attention as insulin has been related to early growth (Madsen, Schack-Nielsen et al. 2010) and breast-feeding was found to be associated with lower risk of obesity and Type 2 diabetes in later life compared with formula feeding (Arenz, Ruckerl et al. 2004; Owen, Martin et al. 2006). Even though differences in long term outcomes between breast and formula fed infants can be related to other differences between human milk and formula, e.g. protein content (Koletzko, von et al. 2009), the differences in plasma glucose and insulin concentrations during the postprandial period depending on the glycaemic index of the meal might also be of importance.

The carbohydrate isomaltulose (Palatinose™) is a low glycaemic (glycaemic index = 32; Atkinson, Foster-Powell et al. 2008) and low insulinaemic carbohydrate. Isomaltulose is, like sucrose, a disaccharide composed of glucose and fructose, but in α-1,6 glycosidic linkage instead of the α-

1,2 linkage in sucrose. Due to the different linkage isomaltulose is only slowly, though completely hydrolyzed by small intestinal disaccharidases and it is a fully available carbohydrate (Lina, Jonker et al. 2002; Holub, Gostner et al. 2010). Thus, isomaltulose does not cause laxation as known from undigestible carbohydrates and provides the full energy content (4 kcal/g). The slower release and absorption of the derived monosaccharides leads to lower postprandial glycaemia and insulinaemia than after oral sucrose administration and the glucose supply is sustained for a longer period of time (Holub, Gostner et al. 2010; van Can, Ijzerman et al. 2009). Isomaltulose is hardly utilized by oral plaque bacteria and is also a toothfriendly carbohydrate (Lina, Jonker et al. 2002).

For these physiological reasons, isomaltulose (Palatinose™) is of interest to be used in infant nutrition to reduce the glycaemic load and/or to develop products that do not promote tooth decay.

Isomaltulose has been used as a dietary sweetener for many years in Japan (Holub, Gostner et al. 2010). The taste and appearance of isomaltulose are similar to sucrose, and the sweetness is about half of that of sucrose. Isomaltulose has been categorized as “generally recognized as safe” (GRAS) in the USA and has been approved as a food component under the Novel Food regulation in the European Union (Holub, Gostner et al. 2010).

Replacement of conventional higher glycaemic carbohydrates like starch, maltodextrin, glucose syrup or sucrose by isomaltulose in infant formulas would make a follow-on formula available with a lowered glycaemic index. The use of such a formula with lowered glycaemic response could lead to attenuated, more balanced postprandial blood glucose and insulin profiles in the recipient infants. This might be more similar to the metabolic and endocrine response observed in breast fed infants. These possible effects might have long-term preventive potential as diet induced enhancement of insulin secretion in infancy has been associated with increased early weight gain as well as higher long-term risk of obesity and associated disorders.

Based on these very encouraging data, the study aims to determine the acceptance and safety of an isomaltulose containing follow-on formula as well as to assess postprandial levels of glucose and insulin at the end of the 4 week intervention period in infants aged at least 5 - 7th months. At this age a large number of infants is no longer breastfed, whereas in many infants complementary feeding has not been introduced yet. This provides the opportunity to detect effects of the formula composition not only on biochemical parameters, but also on infantile behavior.

4. Objectives

The main objectives of the study are to investigate the acceptance, tolerance, and the effect on postprandial glycaemia and insulinemia of a follow-on formula with reduced glycaemic index using isomaltulose (Palatinose™) to partly replace conventional carbohydrates such as starch,

maltodextrin, glucose syrup or sucrose, which are usually high glycaemic, compared to a standing formula. All other components of the two study formulae will be identical.

Specific hypotheses to be tested:

Primary hypothesis:

After 4 week intervention period, insulinemia 60 min after the start of feeding is significantly lower with a formula containing low glycaemic isomaltulose than with a conventional formula containing higher glycaemic carbohydrates.

Secondary hypotheses:

1) after 4 week intervention period, glycaemia 60 min after the start of feeding is significantly lower with a formula containing low glycaemic isomaltulose than with a conventional formula containing higher glycaemic carbohydrates

2) infantile behavior is not different between groups fed a follow-on formula containing low glycaemic isomaltulose or conventional formula

3) parental rating of a follow-on formula containing low glycaemic isomaltulose is not different from the rating of a conventional formula

5. Participating subjects

5.1. Subject number

It is planned to recruit 50 infants at the age of 5 - 7th month of life who will be randomized in two equally sized groups to receive either the low glycaemic formula or a standard formula.

The sample size estimate is based on a study of Lucas et al. (Lucas, Sarson et al. 1980), who found in six day old infants postprandial insulin values of ca. 125 pmol/L in bottle fed and 45 pmol/L in breast fed babies at 90 min post prandium. The standard deviation of the mean was 93.4 pmol/L within a group size of 12 subjects. The observed difference was considered as relevant. So, our sample calculation (power of 0.80; α of 0.05) was aimed to prove a similar difference between isomaltulose- and control-group. The sample size has to be 22 subjects each group, assuming a similar standard deviation. Based on an expected dropout of 15 %, we plan to enroll 25 infants into each group.

5.2 Selection criteria

Inclusion criteria:

- generally healthy infants born at term (37-42 weeks of gestation)

- weight between 10th and 90th percentile for age, according to the EURO Growth guidelines
- age between 5 - 7th month of life at study entry
- fully formula fed for at least 4 weeks before intervention start
- parents/caregivers understand the German language and are able to fill out questionnaires
- parents/caregivers agree to study participation and sign the informed consent form

Exclusion criteria:

- preterm birth (<37 weeks of gestational age)
- acute or chronic illness of infant or mother
- drug and/or alcohol abuses of mother
- chronic medication
- participation of the infant in another intervention study
- gestational diabetes of the mother

Withdrawal criteria

Subjects who meet one or more of the following criteria will be excluded from the per protocol analysis:

- less than 80 % of energy intake is provided by the study formulas during the study period
- medically diagnosed disorders
- subject develops severe adverse event while participating in the study
- parents decide to withdraw their consent to continue participating in the study.

6. Study Procedure

The study is designed as a two-arm, parallel, randomized, double blind, controlled intervention study that will be carried out at Klinikum der Universität München (Universitätsfrauenklinik and Dr. von Hauner Kinderklinik). The infants shall be fully fed with the study formulas for a period of 4 weeks plus maximum 3 working days. Thus, it is planned that the infants consume the study product for a period of about 5 weeks for avoiding abrupt dietary changes. The principle schedule of the study is shown in Figure 1.

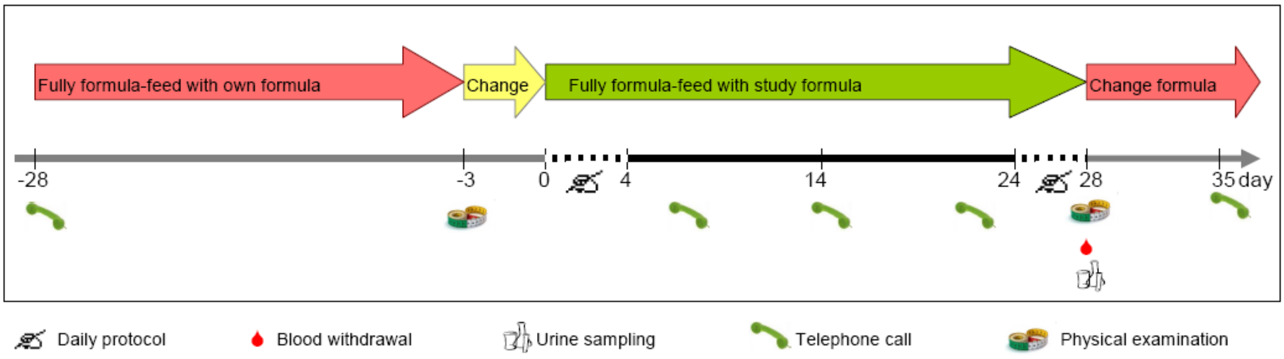

Figure1: Principle schedule of the study for a subject being screened shortly after birth, starting formula feeding 4 weeks before study entry, participates in the pre examination and starts to introduce study formula at age 5 - 7th month of life and completes the study after four weeks with the post examination.

6.1 Screening and Recruitment

Recruitment will be performed at the neonatal department of the Universitätsfrauenklinik, Klinikum der Universität München. After giving birth to healthy babies mothers will be approached during their stay in the hospital, briefly informed about the study and asked for permission to record contact information. In case a mother disagrees no further action will follow and this contact will not be documented. In case she shows interest and provides contact information, the information will be recorded and basic information about the baby (gender, data of birth) will be documented in the screening list. Before the infantile age of 5th month of life the family will be contacted again and asked, whether they are still interested in participation. In case of interest, the study will be explained in detail and it will be discussed, whether a participation is compatible with the plans of the family in respect to feeding their infant during the next months. If this is the case and if no exclusion criteria have become obvious written information about the study will be sent to the family and an appointment for enrolment and baseline examination at the Hauner Childrens Hospital will be made.

Depending on the success of the above mentioned recruitment strategy, additionally recruitment of families of infants aged 1-6 months may be performed in the offices of primary care paediatricians, e.g. at the well baby screening visits (Kindervorsorgeuntersuchungen). Only families of infants that are fed formula will be approached here.

If there is no more interest or other points make a participation impossible the reasons will be recorded in the screening list, but any personal information (especially name, telephone number, and email address) will be permanently deleted.

At the baseline/enrolment visit the parents will have a further possibility to ask questions about the study. If all questions could be satisfactory resolved and if all inclusion criteria are met they will sign the informed consent form. At the enrolment a subject number will be assigned to the infant. These consecutive numbers will be allocated in the sequence of enrolment. Subsequently the baseline examination and data collection will be performed.

6.2 Randomization

A computer generated block randomization list will be established, which assigns subject numbers either to the interventional or the control product. A block size of four is foreseen. Approximately equal distribution of boys and girls between the study groups will be achieved by selecting subject numbers for boys and girls from different blocks. The randomization list will be generated by the sponsor of the study and in cooperation with the formula manufacturer sets of cans with study formula identified with the specific subject number will be produced. These sets will either contain investigational or control formula, thus investigators and parents can identify the allocated formula by the subject number, but are fully blinded towards the group allocation. Sealed envelopes with the formula assignment will be kept for each subject number by the principal investigator. They will only be opened prior unblinding of the study and locking of the study database in case of suspected adverse events, which require knowledge of the composition of the formula fed to the infant. Thus, the randomized formula allocation occurs fully blinded when the subject number is assigned to each subject at enrolment.

6.3. Data collection and intervention

During the **baseline visit** (pre examination) a standardized physical examination of the baby, including anthropometric measures (SOPs, Annex 1), will be performed. Via a standardized interview along the case report forms (CRF, Annex 2) data about the medical history of the baby, information about the pregnancy, previous and current diet of the baby and socioeconomic status of the family will be collected. All information collected during the examination will directly be entered into the CRF for the corresponding subject.

Parents will receive a package of the study formula with the corresponding subject number together with storage and feeding instructions. These instructions will be discussed with the parents, although they should not differ appreciably from standard instructions for formulas in the market. The infant formula fed shall be changed to the study formula within one week after enrolment. During the ensuing study period the infant shall exclusively be fed the study formula (no other infant formulas). Non energy containing liquids can be provided according to parental decision.

Furthermore, the importance of documenting well being and behavior of the infant will be outlined to the parents. They will receive diaries (Annex 3) for documenting the dietary intake and any extraordinary event in respect to the infant from the day of the baseline visit onward and during the 4 weeks period of exclusive feeding until the post intervention examination. A special focus will be put on health related events, e.g. days with increased body temperature. The diary will contain sections for detailed recording of the infant´s day including each feeding (time of the day and amount), regurgitation, vomiting, sleep periods, crying periods, periods of physical activity, stool frequency, and further details. This detailed recording is foreseen for two four days periods with one period during the first week after the infant is fully fed study formula and once during the last study week.

During the intervention period the parents shall only feed the allocated study formula, which they begin to introduce directly after the baseline visit. The concordance of the families will be supported by **weekly telephone calls** to the family from the baseline visit onward. One of these telephone calls will be used to terminate the post intervention examination. In addition they have the possibility to call the study team any time during the study.

The determination of postprandial glucose and insulin will be performed during the **post intervention** visit. It will be attempted to schedule the visit to achieve a 3 hour interval between the last feeding and the next feeding in the study centre (time of the day to be recorded). The physical examination during the post intervention visit will include the same measures as the baseline examination and the anthropometric measures will be taken according to the same procedure. Additionally an urine sample (≥ 3 ml) will be collected via a urine bag, which will be attached during the physical examination.

Three hours after the previous formula feeding, the infant is fed (usually by the parent) a bottle with 120 ml of the study formula assigned to the infant (parents are asked to bring the assigned formula along to the visit). The start and end time of the feeding will be recorded. If the infant does not want to drink the full 120 ml, after 30 min efforts to offer the bottle will be stopped and the amount consumed will be recorded. The infant have to drink 100 ml minimum, otherwise no blood sampling took place and the test will be repeated next day.

A blood sample will be obtained by heel prick 60 min after the start of the feeding. Using a capillary a drop of blood will be applied on a glucose test strip for immediate measurement of full blood glucose. Using a capillary a drop of blood will be applied on a HbA1c kit for immediate measurement of HbA1c level. For the measurement of insulin ≥ 60 µl of blood will be applied on a filter card. After air drying for 4 hours the filter card will be placed in a plastic bag and stored at -80°C for later batch wise analysis. The collected urine will be transferred into a Sarstedt urine monovette (12 ml) and frozen at -80°C.

The parents shall bring the completed diary handed out at the pre examination visit with them and it will be briefly checked for plausibility and if questions arise they will be clarified. The parents will receive a second brief questionnaire for recording the transition from the study formula to a formula of their choice and any health related events during the week following the study, but a further specific follow up is not planned.

6.4. Adverse events (AE)

Within the study special emphasis is put on the detection and recording of adverse events (AE) via the parental diaries and via explicit questions during the post intervention examination.

Any untoward occurrence (including intercurrent diseases, accidents) in a subject participating in the study is considered an AE. The adverse event does not necessarily have to have a causal relationship with the intervention. AE include occasions when the participating families contact the study team or their private physicians and are examined or given medical direction. It may or may not lead to withdrawal of the subjects from the study. All adverse events occurring during the study will be recorded whether or not they are considered to be related to the intervention or not. In case of serious adverse events (SAE, e.g. adverse events, which require hospitalisation, are life-threatening, result in permanent disability/incapacity, an important medical event based upon appropriate medical judgment), these will be reported to the sponsor and the principal Investigator as immediately as possible, independent of the circumstances or suspected cause.

Reporting and documentation of AE has to be done by the investigator on the corresponding page of the CRF (details in Annex 2) and has to be classified as mild, moderate or severe. Documentation shall include: related medical history, diagnosis (if available; otherwise signs or symptoms), duration, maximum intensity, frequency, outcome (AE must be followed until the events are resolved, the condition stabilises, the events are otherwise explained, or the subject is lost to follow-up), actions taken (including change of formula), relationship to intervention (to be judged by the principal investigator considering e.g. temporal coherence between incidence and use of the study formula, influence of removal of study formula on symptoms, observation of the incidence in not study participants, coherence between the incidence and other surrounding conditions, activities).

7. Study diets

The study diet will consist of either the control follow-on formula or the investigational follow-on formula. The investigational formula will provide a portion of its carbohydrates in form of isomaltulose (Palatinose™) rather than higher glycaemic components such as starch, maltodextrin, glucose syrup or sucrose while all other components of the formula will be identical.

The design of the investigational follow-on formula and manufacturing of both formulae will be carried out by an experienced infant formula manufacturer. Before starting the intervention study comprehensive stability and analytical testing (incl. shelf life) will be conducted.

In present study, one meal per day of complementary diet (pureed vegetable or potatoe-vegetable-meat) can feed in addition to study formula, if required. Amount, type and company of complementary diet should documented in diaries.

8. Biochemical analyses

As the amount of blood to be collected has to be kept minimal only measurements of glucose and insulin are planned.

Glucose will be determined in full blood at the time of blood collection using a glucometer, which will be tested rigorously before application for the study. The blood samples will be analysed by the same glucometer.

Insulin will be determined from a dried blood spot (6 mm) applying an ELISA assay specifically adapted for the determination of insulin from dried blood spots by Mercodia (Uppsala, Sweden). Insulin analyses will be conducted in the Institute for Clinical Chemistry of the Klinikum der Universität.

Urinary C-peptide will be measured by an electrochemiluminescence immunoassay and urinary creatinine will be analyzed using the Jaffé reaction by the Institute for Clinical Chemistry of the Klinikum der Universität.

9. Data management and biometrical evaluation

All data will be recorded in specifically prepared CRFs to be incorporated later into an electronic database. In the database they will be combined with the results of the laboratory analyses and the data collected via parental diaries and questionnaires. Quantitative data will be introduced as measured into the database, while response to questions will be categorized either already at the time of documentation (only a defined selection of responses allowed) or in cases where this is not feasible categorization will be made at the data entry, as far as possible.

For all quantitative parameters determined a test for normal distribution will be performed according to Kolmogorov-Smirnov. If normal distribution can be assumed, mean and standard deviation will be given for these parameters. For non normally distributed data median and interquartile range will be provided and data will be transformed appropriately prior to statistical analysis.

For the statistical analysis of the specific hypotheses initially results of the groups will be compared using Students t-test, Mann Whitney-test or Chi^2-test as appropriate. Within the hypotheses testing a correction for multiple testing will not generally be applied, as each of the hypotheses is tested independently, but if more than one parameter is available (e.g. safety) a post hoc correction according to Bonferroni will be made.

For this primary analysis a distinction will be made between subjects, who completed the study according to protocol (per-protocol group) and those, who only qualify for the intention to treat analysis. The analysis will be performed for both groups of subjects. The distinction between the groups will be based on the parentally reported formula intake: infants, who obtain at least 80 % of their energy intake from the study formula, qualify for the per- protocol group. If it is considered appropriate, a lower percentage may be chosen for the per-protocol group in agreement with the sponsor.

To test the primary hypothesis Student’s t-test will be performed and if necessary linear regression is applied including confounding factors for which randomization failed. The first of the secondary hypothesis will be tested accordingly. For the second and third secondary hypotheses questionnaire data will be categorized and rated to get ordinal outcomes. Those will be compared between the two groups using Mann-Whitney-U-tests.

The initial database will be set up in Microsoft Excel, but for the statistical analysis STATA or SPSS will be applied.

10. Ethical consideration, safety and insurance

The study will be conducted in accordance with the Declaration of Helsinki and the Good Clinical Practice guidelines. Written informed consent will be obtained from the infant’s parents as an obligatory requisite to include the subject in the study (Annex 4). No procedures related to this study will be carried out before the consent form is duly signed and dated. The subject information will contain the study purpose, the description of how and for how long the child and parents will be participating in the study, the content and characteristics of the study formulas, the potential risks and benefits of participating in the study and the assurance of preserving confidentiality of the data obtained and anonymity of participants in the study. An adequate amount of time will be given to the parents to carefully read it, ask questions and finally decide if they accept to participate or not. If accepted, parents sign the Consent Form after hand-writing their name and date. Approval of the Study Protocol, subject information and consent form will be sought from the Ethical Committee at LMU München before initiation of the study.

Although no risks in context with study participation are anticipated an insurance of the clinical trial will be conducted, which financially compensates for any damage of the participating infants related to the study participation independent of proven liability.

The participating families will receive financial compensation for their expenses in relation to study participation and incentives will be provided. In addition, the infant will receive a commercial follow-on formula for additional 6 months to avoid the infants have to change their nutrition again. This follow-on formula will be the same formula like the control formula used in the study (commercial formula with high-glycaemic carbohydrates).

11. References

Arai, H., A. Mizuno, et al. (2007). "Effects of a palatinose-based liquid diet (Inslow) on glycemic control and the second-meal effect in healthy men." Metabolism **56**(1): 115-121.

Arenz, S., R. Ruckerl, et al. (2004). "Breast-feeding and childhood obesity - a systematic review." Int.J Obes.Relat Metab Disord. **28**(10): 1247-1256.

Atkinson, F. S., K. Foster-Powell, et al. (2008). "International tables of glycemic index and glycemic load values: 2008." Diabetes Care **31**(12): 2281-2283.

Bornet, F. R., A. E. Jardy-Gennetier, et al. (2007). "Glycaemic response to foods: impact on satiety and long-term weight regulation." Appetite **49**(3): 535-553.

Fujiwara, T., Y. Naomoto, et al. (2007). "Effects of a novel palatinose based enteral formula (MHN-01) carbohydrate-adjusted fluid diet in improving the metabolism of carbohydrates and lipids in patients with esophageal cancer complicated by diabetes mellitus." J Surg Res **138**(2): 231-240.

Holub, I., A. Gostner, et al. (2010). "Novel findings on the metabolic effects of the low glycaemic carbohydrate isomaltulose (Palatinose (TM))." British Journal of Nutrition **103**(12): 1730-1737.

Koletzko, B., K. R. von, et al. (2009). "Lower protein in infant formula is associated with lower weight up to age 2 y: a randomized clinical trial." Am J Clin Nutr **89**(6): 1836-1845.

Lina, B. A., D. Jonker, et al. (2002). "Isomaltulose (Palatinose): a review of biological and toxicological studies." Food Chem.Toxicol. **40**(10): 1375-1381.

Lucas, A., S. Boyes, et al. (1981). "Metabolic and endocrine responses to a milk feed in six-day-old term infants: differences between breast and cow's milk formula feeding." Acta Paediatr.Scand. **70**(2): 195-200.

Lucas, A., D. L. Sarson, et al. (1980). "Breast vs bottle: endocrine responses are different with formula feeding." Lancet **1**(8181): 1267-1269.

Madsen, A. L., L. Schack-Nielsen, et al. (2010). "Determinants of blood glucose and insulin in healthy 9-month-old term Danish infants; the SKOT cohort." Diabetic Medicine **27**(12): 1350-1357.

Oh, K., F. B. Hu, et al. (2005). "Carbohydrate intake, glycemic index, glycemic load, and dietary fiber in relation to risk of stroke in women." Am J Epidemiol. **161**(2): 161-169.

Okuno, M., M. K. Kim, et al. (2010). "Palatinose-blended sugar compared with sucrose: different effects on insulin sensitivity after 12 weeks supplementation in sedentary adults." Int J Food Sci Nutr **61**(6): 643-651.

Owen, C. G., R. M. Martin, et al. (2006). "Does breastfeeding influence risk of type 2 diabetes in later life? A quantitative analysis of published evidence." American Journal of Clinical Nutrition **84**(5): 1043-1054.

Reynolds, R. C., K. S. Stockmann, et al. (2009). "Effect of the glycemic index of carbohydrates on day-long (10 h) profiles of plasma glucose, insulin, cholecystokinin and ghrelin." European Journal of Clinical Nutrition **63**(7): 872-878.

Salmenpera, L., J. Perheentupa, et al. (1988). "Effects of Feeding Regimen on Blood-Glucose Levels and Plasma-Concentrations of Pancreatic Hormones and Gut Regulatory Peptides at 9 Months of Age - Comparison between Infants Fed with Milk Formula and Infants Exclusively Breast-Fed from Birth." Journal of Pediatric Gastroenterology and Nutrition **7**(5): 651-656.

van Can, J. G., T. H. Ijzerman, et al. (2009). "Reduced glycaemic and insulinaemic responses following isomaltulose ingestion: implications for postprandial substrate use." Br.J Nutr **102**(10): 1408-1413.

Wallensteen, M., B. S. Lindblad, et al. (1991). "Acute C-Peptide, Insulin and Branched-Chain Amino-Acid Response to Feeding in Formula and Breast Fed Infants." Acta Paediatrica Scandinavica **80**(2): 143-148.

Zhang, C., S. Liu, et al. (2006). "Dietary fiber intake, dietary glycemic load, and the risk for gestational diabetes mellitus." Diabetes Care **29**(10): 2223-2230.
