# Supplementary material for: Effects of a Follow-On Formula Containing Isomaltulose (Palatinose™) on Metabolic Response, Acceptance, Tolerance and Safety in Infants: A Randomized-Controlled Trial
Source: PLoS One. 2016 Mar 17;11(3):e0151614. doi: 10.1371/journal.pone.0151614 (PMC4795687; doi:10.1371/journal.pone.0151614)
Supplement: S2 Text — (PDF) [file pone.0151614.s005.pdf]

beneo institute
connecting nutrition and health

NSC, 29.11.2012
Carolin Sieland , 12006n-CSD-CSD
+49 (0) 6359/803 831

Amendment Nr. 1 to the study protocol[1] of the study

***"Safety, acceptance and metabolic effects in infants receiving a novel low glycaemic index follow-on formula"* ("AMELIE" study)**

Following arrangements and protocol modifications have been agreed between investigators and the sponsor:

- Chapter 10 of the study protocol states: "*The participating families will receive financial compensation for their expenses...[]. In addition, the infant will receive a commercial follow-on formula for additional 6 months to avoid the infants have to change their nutrition again. This follow-on formula will be the same formula like the control formula used in the study (commercial formula with high glycaemic carbohydrates).*" This provision is not possible and therefore no longer foreseen.

[redacted]

We consent to the above research protocol changes and arrangements:

Investigators:

Munich, 13.12.12 ____________

Prof. Dr. Berthold Koletzko

(Principle investigator)

BENEO (sponsor):

Obrigheim, Nov 29th 2012 ____________ ____________

Dr. Stephan Theis   Carolin Sieland[2]

[1] Final study protocol version February 6th 2012 (Study_Protocol_AMELIE_060212.doc)

[2] New contact person on behalf of sponsor replacing Ines Holub

Klinikum der Universität München · Kinderklinik und Kinderpoliklinik im Dr. von Haunerschen Kinderspital, Lindwurmstr. 4, 80337 München

Dr. von Haunersches Kinderspital

Univ.-Prof. Dr. Berthold Koletzko
Sekretariat: Birgit Kessler
Telefon +49 (0)89 5160 - 2826
Telefax +49 (0)89 5160 - 7742
office.koletzko@med.uni-muenchen.de

www.klinikum.uni-muenchen.de

Postanschrift:
Lindwurmstraße 4
80337 München

Ihr Zeichen: Unser Zeichen: München, 04.03.2013

Amendment Nr. 2 to the study protocol of the study

„Safety, acceptance and metabolic effects in infants receiving a novel low glycaemic index follow-on formula" ("AMELIE" study)

Following arrangements and protocol modifications have been agreed between investigator and sponsor:

- The Inclusion criteria of Chapter 5.2 "Selection criteria" states:
  - weight between 10th and 90th percentile for age, according to the EURO Growth guidelines

    ➔ Instead, weight between 5th and 95th percentile for age, according to the EURO Growth guidelines will be allowed. An influence on study outcome and estimated statistical power is not expected.

- The Inclusion criteria of Chapter 5.2 "Selection criteria" states:
  - fully formula fed for at least 4 weeks before intervention start

    ➔ Instead, at least 2 weeks of full formula feeding before intervention start will be allowed. Thus, infants whose parents are interested in the study, but want their babies to be fully breastfed for 6 months, according to the guidelines of the WHO, can be included into the study within the given acceptable age range of the infants.

[redacted]

We consent to the above protocol changes:

Investigator:

Munich, 5.3.13

Prof. Dr. Berthold Koletzko

(Principle investigator)

BENEO (sponsor):

Obrigheim, 20.3.13

Dr. Stephan Theis

Carolin Sieland

Klinikum der Universität München · Kinderklinik und Kinderpoliklinik im Dr. von Haunerschen Kinderspital, Lindwurmstr. 4, 80337 München

Dr. von Haunersches Kinderspital

Univ.-Prof. Dr. Berthold Koletzko
Sekretariat: Birgit Kessler
Telefon +49 (0)89 5160 - 2826
Telefax +49 (0)89 5160 - 7742
office.koletzko@med.uni-muenchen.de

www.klinikum.uni-muenchen.de

Postanschrift:
Lindwurmstraße 4
80337 München

Ihr Zeichen: Unser Zeichen: München, 18.06.2013

Amendment Nr. 3 to the study protocol of the study

„Safety, acceptance and metabolic effects in infants receiving a novel low glycaemic index follow-on formula" ("AMELIE" study)

Following arrangements and protocol modifications have been agreed between investigator and sponsor:

- The Inclusion criteria of Chapter 5.2 "Selection criteria" states:
  - age between 5 - 7th month of life at study entry
    - ➔ Instead, age between 5th and 9th month of age will be allowed. The common reference values of insulin and glucose do not differ in infants between 7 and 9 months. Thus, an influence on study outcome is not expected.
- The dietary regime of Chapter 7 "Study diet".
  - One meal per day of complementary diet (pureed vegetable or potatoe-vegetable-meat) can feed in addition to study formula
    - ➔ Instead, the kind of complementary feeding, e.g. vegetable or fruits given to the infants can be chosen freely. But the amount of complementary feeding is restricted to a maximum 25% of

energy intake and to a maximum of 25% of carbohydrate intake. Furthermore, the infants are not allowed to have complementary feeding 6 hours before the final visit.

The introduction of complementary feeding according to the national recommendations may occur starting with vegetable-/vegetable-meat, followed by milk-cereal and then fruit-mush. With increasing study age, there will be more variance in kind and amount of complementary feeding. Similar to another study (Picaud et al. 2010), a maximum of 25% of other food than study formula is allowed. The energy and carbohydrate intakes via complementary feeding are calculated for each subject individually and are restricted to a maximum of 25% of total energy or carbohydrate intake.

Infants should not eat complementary feeding within a time interval of 6 hours before the final study visit to avoid any undue of a high carbohydrate intake e.g. from fruit mush on study outcome measures.

We consent to the above protocol changes:

Investigator:

Munich, 23.06.13

Prof. Dr. Berthold Koletzko
(Principle investigator)

BENEO (sponsor):

Obrigheim, 01.07.13

Dr. Stephan Theis

Carolin Sieland
